# Back-propagation neural network-based reconstruction algorithm for diffuse optical tomography

**DOI:** 10.1117/1.JBO.24.5.051407

**Published:** 2018-12-19

**Authors:** Jinchao Feng, Qiuwan Sun, Zhe Li, Zhonghua Sun, Kebin Jia

**Affiliations:** a Beijing University of Technology, Beijing Key Laboratory of Computational Intelligence and Intelligent System, Faculty of Information Technology, Beijing, China; b Beijing Laboratory of Advanced Information Networks, Beijing, China

**Keywords:** diffuse optical tomography, back-propagation neural network, image reconstruction, inverse problem

## Abstract

Diffuse optical tomography (DOT) is a promising noninvasive imaging modality and is capable of providing functional characteristics of biological tissue by quantifying optical parameters. The DOT image reconstruction is ill-posed and ill-conditioned, due to the highly diffusive nature of light propagation in biological tissues and limited boundary measurements. The widely used regularization technique for DOT image reconstruction is Tikhonov regularization, which tends to yield oversmoothed and low-quality images containing severe artifacts. It is necessary to accurately choose a regularization parameter for Tikhonov regularization. To overcome these limitations, we develop a noniterative reconstruction method, whereby optical properties are recovered based on a back-propagation neural network (BPNN). We train the parameters of BPNN before DOT image reconstruction based on a set of training data. DOT image reconstruction is achieved by implementing a single evaluation of the trained network. To demonstrate the performance of the proposed algorithm, we compare with the conventional Tikhonov regularization-based reconstruction method. The experimental results demonstrate that image quality and quantitative accuracy of reconstructed optical properties are significantly improved with the proposed algorithm.

## 1 Introduction

Diffuse optical tomography (DOT) has shown a great potential for breast imaging^1^^–^^9^ and functional brain imaging,^10^^–^^12^ which use near-infrared light in the spectral range of 600 to 950 nm to quantify tissue optical (absorption and scattering) coefficients. There is a critical need to develop an efficient image reconstruction algorithm for DOT. Recovering the internal distribution of optical properties is a severely ill-posed and under-determined inverse problem, due to light propagation in highly scattering biological tissues and limited number of measurements,^13^^,^^14^ which makes image reconstruction challenging.

Although both linear and nonlinear reconstruction algorithms for DOT are available,^14^ considerable efforts have been made to develop various reconstruction algorithms to improve quantitative accuracy and image quality.^14^^–^^22^ To date, the ill-posedness of the inverse problem in DOT can be alleviated by employing a regularization technique, which utilizes a data fitting term together with a regularizer ($L_{2}$ or $L_{1}$ norm, etc.) to suppress the effect of measurement noise and modeling errors.^23^

When the regularizer is a $L_{2}$ norm, the reconstruction algorithm becomes the well-known Tikhonov regularization method, which imposes restrictions on the $L_{2}$ norm of the optical properties^23^ and is optimal when the distribution of optical properties subjects to Gaussian distribution.^24^ The merits of Tikhonov regularization are simple, and easy to be implemented. However, the $L_{2}$ norm will oversmooth reconstructed images and yield low-quality images by penalizing large values.^23^ An alternative regularizer is total variation (TV) norm, which is the ideal choice when the distribution of optical properties is known to be piecewise constant.^24^ Another possible regularizer is Lp norm (0<p<=1), which poses a sparsity constraint on the optical properties. The quality of reconstructed images can be improved with the use of sparsity regularization.^23^^,^^25^

The abovementioned algorithms are generally iterative reconstruction algorithms.^14^ These algorithms require heavy computation and large storage memory because the forward problem must be solved repeatedly, and an updated distribution of optical properties must be found at each iterative step.^14^ However, iterative reconstruction algorithms have limited capability in terms of reconstruction accuracy and image quality,^26^ which are important for accurate diagnosis of breast cancers. In addition, how to accurately choose parameters, particularly regularization parameter, for iterative reconstruction algorithms needs to be further considered.^27^

Recently, artificial neural networks with various network architectures, including deep convolutional neural network,^28^^,^^29^ generative adversarial networks,^30^ and multilayer perceptron,^31^ have achieved significant improvements over existing iterative reconstruction methods in the quality of reconstructed images. It is likely that image recovery in DOT benefits from these important developments.

In this work, we investigated the feasibility and effectiveness of a back-propagation (BP) neural network (BPNN) to recover the distribution of optical properties in DOT. BPNN is a widely used neural network because it has many advantages. For example, BPNN is simple, efficient at computing the gradient descent, and straightforward to implement. The basic procedure of BPNN includes the forward propagation of input data and the reverse transmission of output error.^32^ More detailed introduction about BPNN can be found in Refs. 33–39. The forward propagation of input data is to transmit input data from the input layer through a series of hidden layers toward the output layer, which builds the relationship between input data and output data. The reverse transmission of output error between the calculated and the ground true output is backward propagated from the output layer through the hidden layers to the first layer to adjust the connection weights and bias variables of neurons. By repeatedly applying this procedure, the output error is adjusted to an expected range. We validate the proposed method using simulation experiments and compare BPNN with the popular Tikhonov regularization. Our results demonstrate that our method provides higher accuracy and superior image quality than Tikhonov regularization in recovering a single inclusion or two closely spaced inclusions.

The remainder of the paper is organized as follows. Section 2 describes the light propagation model, BPNN, and evaluation metrics. Experimental results and comparisons are presented in Sec. 3. Finally, we present a discussion of results with our conclusions and future work in Sec. 4.

## 2 Methods

### 2.1 Forward Model

The light propagation in biological tissues can be modeled by the steady diffusion equation,^13^^,^^14^ which can be described as follows:

$$-\nabla \cdot D(r)\nabla \Phi (r)+\mu_{a}(r)\Phi (r)=q_{0}(r),\;(r\in \Omega ),$$ (1)

where Ω is the imaged object, Φ(r) is the photon fluence rate at position r, $D=1/[3*(\mu_{a}+\mu_{s}^{\prime })]$ is the diffusion coefficient (${\mathrm{mm}}^{-1}$), $\mu_{a}$ is the absorption coefficient (${\mathrm{mm}}^{-1}$), $\mu_{s}^{\prime }$ is the reduced scattering coefficients (${\mathrm{mm}}^{-1}$), and $q_{0}(r)$ is the source term.

Here, the boundary condition used for Eq. (1) is Robin-type condition, which can be expressed as follows:^13^^,^^14^

Φ(r)+Dαn^·∇Φ(r)=0(r∈∂Ω), (2)

where ∂Ω is the surface boundary of imaged object Ω, α is the boundary mismatch parameter, and n^ is the outer normal on ∂Ω.

When the distributions of $\mu_{a}$ and D are known, light measurements at the detectors can be calculated by solving Eqs. (1) and (2) based on the finite-element method,^40^ which can be modeled with the following equation:

$$f(x)=\Phi^{m},$$ (3)

where $x\in R^{2N}$ and $\Phi^{m}\in R^{M}$ represent the optical properties ($\mu_{a}$ and $\mu_{s}^{\prime }$) and the measurements at the detectors, respectively; N and M are the number of finite-element nodes and the number of boundary measurements, respectively; and f(·) is the forward operator that relates the unknown distribution of optical properties to the boundary measurements.

### 2.2 Back-Propagation Neural Network-Based Reconstruction

**Fig. 1:**
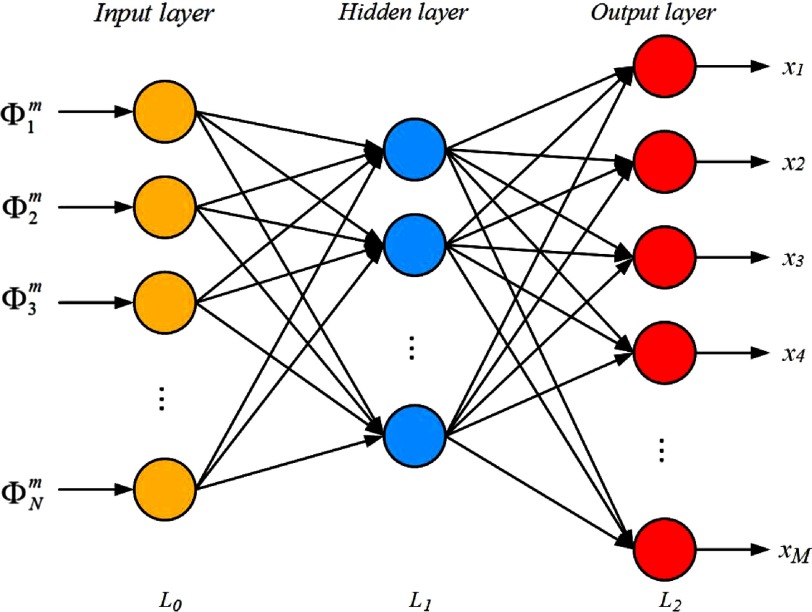
The architecture of the three-layer BPNN used in DOT image reconstruction.

To improve the performances of iterative reconstruction algorithms in DOT, here we develop a reconstruction algorithm based on a BPNN. BPNN is divided into three types of layers: the input layer ($L_{0}$), the fully connected hidden layer ($L_{1}$), and the predictable output layer ($L_{2}$). The architecture of the three-layer BPNN used for DOT image reconstruction is shown in Fig. 1. We train the neural network from the boundary measurements to learn a DOT reconstruction. In this work, the reduced scattering coefficient ($\mu_{s}^{\prime }$) is assumed to be spatially constant and known and we recover only the absorption coefficient ($\mu_{a}$). Therefore, for the network training, boundary measurements $\Phi_{i}(i=1,\ldots ,M)$ (i.e., amplitude) are regarded as the input vector, which are generated by solving the forward model using open source software Nirfast,^40^ and the ground true distribution of absorption coefficient $x_{j}(j=1,\ldots ,N)$ is served as the expected output.

During the training process, the error between the predicted output and the true output is backward propagated from the output layer to the hidden layer to adjust the weights and biases in the opposite direction to the signal flow with respect to each individual weight.^32^ By repeatedly applying this procedure for each sample in the training set, the learning process can converge.

The BPNN-based reconstruction algorithm can be described as follows:^36^

Step 1: Randomly initialize the weights $w_{ij}^{\left[l\right]}$ and the bias variables $v_{j}^{\left[l\right]}$ at the l’th (l=1,2) layer, and set the stop threshold δ and maximum number of iteration $n_{\max}$;
Step 2: Compute the output $o_{j}^{\left[l\right]}$ of the j’th neuron at the l’th (l=1,2) layer using Eq. (4):
  $$o_{j}^{\left[l\right]}=g(\overset{N^{\left[l\right]}}{\underset{i=1}{\sum }}w_{ij}^{\left[l\right]}y_{i}^{\left[l-1\right]}+v_{j}^{\left[l\right]}),$$ (4)
  where g(·) is the neuron activation function, $y^{\left[l\right]}$ is the input of the l’th layer, $N^{\left[l\right]}$ is the number of the l’th layer neurons. Here, $y^{\left[0\right]}$ is the input of the network, i.e., $\Phi^{m}$.
Step 3: Calculate the mean square error for the output layer (l=2) between the ground truth output $d_{j}$ and the predicted output $o_{j}$ according to Eq. (5):
  $${\mathrm{err}}_{j}^{\left[2\right]}=-g^{\prime }(o_{j}^{\left[2\right]})(d_{j}-o_{j}^{\left[2\right]})\;(j=1,\ldots ,N^{\left[2\right]})$$ (5)
  and compute the mean square error in the hidden layer (l=1) using Eq. (6):
  $${\mathrm{err}}_{j}^{\left[1\right]}=g^{\prime }(o_{j}^{\left[1\right]})\overset{N^{\left[2\right]}}{\underset{k=1}{\sum }}{\mathrm{err}}_{k}^{\left[2\right]}w_{jk}^{\left[2\right]},$$ (6)
  where $g^{\prime }(\cdot )$ is the derivative of neuron activation function.
Step 4: Adjust the connection weights and biases between layers at the n’th iteration based on Eqs. (7) and (8):
  $$v_{ij}^{\left[l\right]}(n+1)=v_{i}^{\left[l\right]}(n)+\eta \cdot {\mathrm{err}}_{j}^{\left[l\right]}$$ (7)
  $$w_{ij}^{\left[l\right]}(n+1)=w_{ij}^{\left[l\right]}(n)+\eta \cdot {\mathrm{err}}_{j}^{\left[l\right]}\cdot o_{i}^{\left[l-1\right]}$$ (8)
  where η is the learning rate, which controls the speed of adjusting neural network weights based on the gradient descent method.
Step 5: Go back to Step 2 if the mean squared error of the neural network output is larger than the given stop threshold δ, or n is smaller than $n_{\max}$, or the loss function is less than ε; otherwise, the training processing will be terminated and output the weights and biases.
Step 6: Directly reconstruct the distribution of absorption coefficient by evaluating the trained network.

In our algorithm, the Tansig function is adopted as the activation function. Its formula is given in Eq. (9):

$$g(x)=\mathrm{tansig}(x)=\frac{e^{x}-e^{-x}}{e^{x}+e^{-x}},$$ (9)

and its derivative is shown in Eq. (10):

$$g^{\prime }(x)=1-{\left[g(x)\right]}^{2}.$$ (10)

### 2.3 Evaluation Metrics

The performance of the proposed algorithm is accessed with four evaluation metrics, including the absolute bias error (ABE), mean square error (MSE),^41^^,^^42^ peak signal-to-noise ratio (PSNR),^43^ and structural similarity index (SSIM).^44^ These parameters are defined as follows:

$$\mathrm{ABE}=\frac{\sum_{i=1}^{N}|x_{\mathrm{true}(i)}-x_{\mathrm{recon}(i)}|}{N},$$ (11)

$$\mathrm{Var}=\frac{\sum_{i=1}^{N}|x_{\mathrm{true}(i)}-{\bar{x}}_{\mathrm{recon}}|}{N},$$ (12)

$$\mathrm{MSE}={\mathrm{ABE}}^{2}+\mathrm{Var},$$ (13)

$$\mathrm{PSNR}=10\;\log_{10}\{\frac{{\left[\max(x_{\mathrm{recon}})\right]}^{2}}{\mathrm{MSE}}\},$$ (14)

$$\mathrm{SSIM}=\frac{\left(2{\overset{¯}{x}}_{\mathrm{true}}{\overset{¯}{x}}_{\mathrm{recon}}+c_{1})(2\sigma_{\mathrm{true},\mathrm{recon}}+c_{2}\right)}{\left({\overset{¯}{x}}_{\mathrm{recon}}^{2}+{\overset{¯}{x}}_{\mathrm{recon}}^{2}+c_{1})(\sigma_{\mathrm{true}}^{2}+\sigma_{\mathrm{recon}}^{2}+c_{2}\right)},$$ (15)

where $x_{\mathrm{true}(i)}$ and $x_{\mathrm{recon}(i)}$ are the true and reconstructed absorption coefficients at the finite node i, respectively; ${\overset{¯}{x}}_{p}(p=\text{true or recon})$ and $\sigma_{p}(p=\text{true or recon})$ are the mean and standard derivation for the ground true (p=true) or reconstructed (p=recon) absorption coefficients, respectively; $\sigma_{\mathrm{true},\mathrm{recon}}$ is the covariance between the ground true and the reconstructed absorption coefficients, and $c_{1},c_{2}$ are stabilization constants used to prevent division by a small denominator;^44^ N is the above mentioned number of finite-element nodes. The ABE and MSE are used to compare the accuracy of reconstructed images. The PSNR (unit: dB) is used to compare the restoration of the images, without depending strongly on the image intensity scaling.^15^ SSIM is used to measure the similarity between the true and the reconstructed images, and an SSIM value of 1.0 refers to identical images. We expect lower ABE and MSE, while higher PSNR and SSIM, which show better performance.

## 3 Results

### 3.1 Data Preparation

**Fig. 2:**
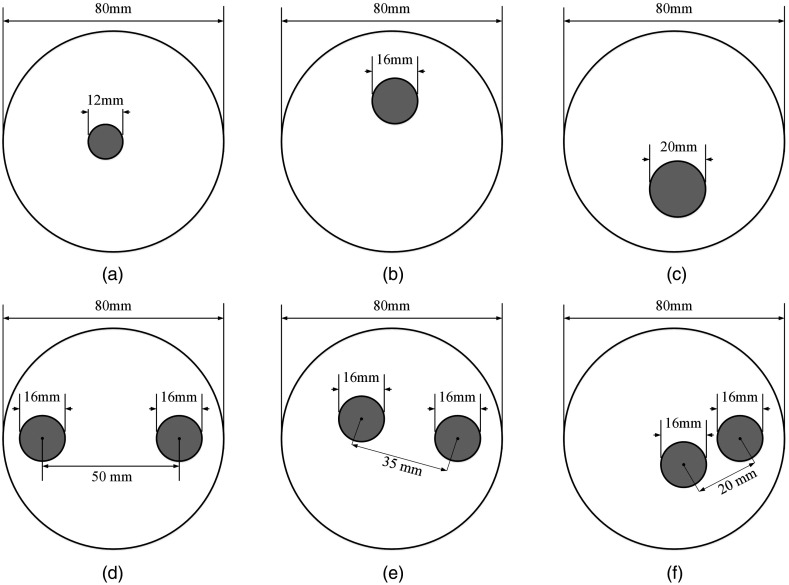
Examples of geometries of generating datasets. (a)–(c) The examples of geometry with a single inclusion that has different locations and sizes. (d)–(f) The examples of geometries with two inclusions that have different edge-to-edge distance and locations. The absorption coefficients of inclusions in each geometry are varied from 0.015 to $0.08\;{\mathrm{mm}}^{-1}$.

A 2-D circular phantom with a diameter of 80 mm was used to generate dataset. It was discretized into 2001 finite-element nodes and 3867 triangular elements. The absorption coefficient ($\mu_{a}$) and the reduced scattering coefficient ($\mu_{s}^{\prime }$) of the phantom were $0.01\;{\mathrm{mm}}^{-1}$ and $1.0\;{\mathrm{mm}}^{-1}$, respectively. A total of 16 sources and 16 detectors were uniformly arranged along the circumference of the phantom. For each source illumination, data were collected at the remaining 15 detector locations, thus leading to a total of 240 (16×15) measurements. To generate simulation datasets, the phantom includes circular inclusions associated with varied sizes, locations, and absorption coefficients. Initially, an inclusion with the diameter of 6, 8, or 10 mm was randomly placed at different locations over the phantom. In this case, the absorption coefficients of the inclusions were varied from 0.015 to $0.08\;{\mathrm{mm}}^{-1}$, leading to 17075 geometries. Next, two inclusions, which have the same radius of 8 mm, were placed at different edge-to-edge distances over the phantom. In this case, the phantoms were assigned different absorption coefficients (0.015, 0.02, 0.04, 0.06, or $0.08\;{\mathrm{mm}}^{-1}$) to each of its inclusions, leading to 5015 geometries. In all cases, the reduced scattering coefficients of inclusions were assumed to be $1\;{\mathrm{mm}}^{-1}$, which were the same as those of the background. Examples of geometries of generating data are shown in Fig. 2. Software Nirfast was used to generate the simulation data,^40^ and 2% random Gaussian noise was added to the measurement data. A total of 22,090 samples which are data pairs contained input data and desired output data were obtained, and were separated into training, validation, and testing datasets. About 20,000 samples were used for training, 1045 for validation, and 1045 for testing.

In the following experiments, the neural network has three layers: an input layer (240 neurons), a fully connected hidden layer, and an output layer (2001 neurons). To determine the number of neurons in the hidden layer, an empirical formula that has been introduced in Ref. 45 was used. The formula is given by

$$s=\sqrt{m(n+2)}+1,$$ (16)

where s is the number of the hidden layer units, m and n are the numbers of input neurons and output neurons. In our experiments, the values of m and n are 240 and 2001, respectively. According to Eq. (16), the calculated number of neurons in hidden layer is 694.3. In our experiments, the number of neurons in the hidden layer was set to 695. The learning rate η, the stopping threshold δ, the maximum number of iteration and the threshold ε were set to be 10, $1\times 10^{-5}$, 50,000 and $2\times 10^{-5}$, respectively. BPNN was trained for 16,000 epochs to minimize the MSE between the true and the recovered images. It took about 26 h to train the BP neural network. The experiments ran on a personal computer with Intel Core i7 CPU at 2.8 GHz and 8 GB RAM.

For the purpose of comparison, we also performed Tikhonov regularization-based DOT reconstruction based on the Nirfast software.^40^ Tikhonov regularization-based DOT reconstruction is achieved using a least squares (LS) minimization technique, which is solved in the Levenberg–Marquardt procedure.^40^ The objective function in the Tikhonov regularization-based reconstruction algorithm typically consists of a data fidelity term of weighted LS and a regularization term of $L_{2}$ norm, balanced by a regularization parameter λ. For λ at the k’th iteration, it was setting as $\lambda_{k}=10*\max[\mathrm{diag}(J_{k}^{T}J_{k})]$, where $J_{k}$ is the Jacobian matrix at the k’th iteration. The stopping criterion for Tikhonov regularization is defined such that the algorithm stops when the change in the difference between the forward data and the reconstructed data of two successive iterations is less than 2% or the maximum number of iteration (50) is reached. The initial regularization parameter is set to be 10. More detailed information about Tikhonov regularization-based DOT reconstruction can be found in Ref. 40.

### 3.2 Experimental Results

**Fig. 3:**
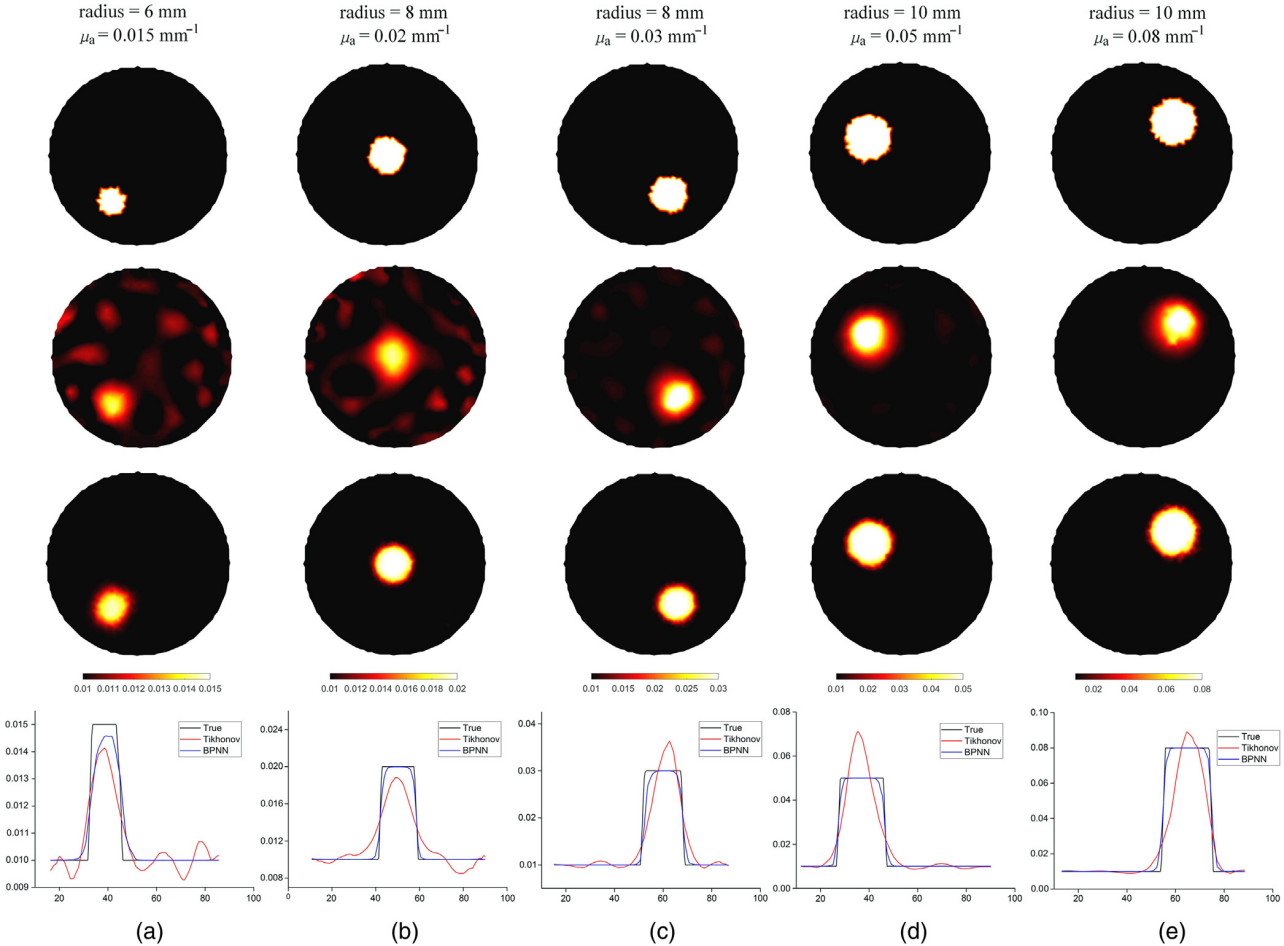
(a)–(e) Reconstructed images of a single inclusion with different sizes and different values of absorption coefficients. The first row is the true images, the second and third rows are the recovered images using Tikhonov regularization and BPNN, respectively. The last row is the corresponding profiles through the center of inclusions and along x axis. The sizes and the true values of absorption coefficients for each case are shown at the top of the figure. The reconstructed images of each column are shown at the same scale.

In this subsection, we provide numerical simulations to illustrate recovered results using BPNN and compare it with widely used Tikhonov regularization-based reconstruction method. Figure 3 shows some examples of recovered absorption coefficient using the two algorithms in the case of single inclusion. In Fig. 3, the size, the location, and the absorption coefficient of inclusions are varied. The ground true images are shown in the top row of Fig. 3, the reconstructed images using Tikhonov regularization and BPNN are given in the second and the third rows of Fig. 3, respectively. The corresponding cross-section profiles through their centers of the inclusions and along the x axis are plotted in the last row of Fig. 3. From the last row of Fig. 3, we can see that the maximum values of recovered $\mu_{a}$ using Tikhonov regularization are much higher compared to their ground truths, the recovered $\mu_{a}$ using BPNN matched with their true values. The quantitative comparisons for the five cases in Fig. 3 are listed in Table 1. Compared with Tikhonov regularization, the values of ABE and MSE obtained using BPNN are significantly reduced, and the values of PSNR and SSIM are greatly improved. As an example, the values of ABE and MSE in Fig. 3(a) are reduced by 80% and 61%, respectively, which show that BPNN can yield higher reconstruction accuracy. It is evident that BPNN provides high-quality images with less artifacts in the background than those of Tikhonov regularization. Therefore, BPNN can have a PSNR gain of about 4.4 dB over Tikhonov regularization while the higher value of SSIM is obtained. The value of SSIM is improved by 424%, which indicates that the recovered image is nearly the same with the ground truth image. The similar results can also be observed in other images of Fig. 3. These results show that BPNN outperforms Tikhonov regularization in terms of higher accuracy and better image quality. In addition, the results also show that the image quality obtained by Tikhonov regularization is improved with increment of size and absorption coefficient of inclusions. By contrast, BPNN can always obtain robust reconstruction results.

**Table 1:** The quantitative comparisons presented in Fig. 3.

Metric	Method	Fig. 3(a)	Fig. 3(b)	Fig. 3(c)	Fig. 3(d)	Fig. 3(e)
ABE	Tikhonov	$4.35\times 10^{-4}$	$8.52\times 10^{-4}$	$8.70\times 10^{-4}$	$1.69\times 10^{-3}$	$2.50\times 10^{-3}$
	BPNN	$8.51\times 10^{-5}$	$1.00\times 10^{-4}$	$1.77\times 10^{-4}$	$4.38\times 10^{-4}$	$8.60\times 10^{-4}$
MSE	Tikhonov	$3.75\times 10^{-7}$	$1.62\times 10^{-6}$	$3.35\times 10^{-6}$	$2.06\times 10^{-5}$	$6.19\times 10^{-5}$
	BPNN	$1.45\times 10^{-7}$	$2.30\times 10^{-7}$	$9.78\times 10^{-7}$	$5.63\times 10^{-6}$	$1.90\times 10^{-5}$
PSNR (dB)	Tikhonov	27.37	23.43	26.10	24.44	21.58
	BPNN	31.75	32.40	29.64	26.47	25.28
SSIM	Tikhonov	0.17	0.20	0.45	0.75	0.84
	BPNN	0.89	0.97	0.96	0.96	0.96

**Fig. 4:**
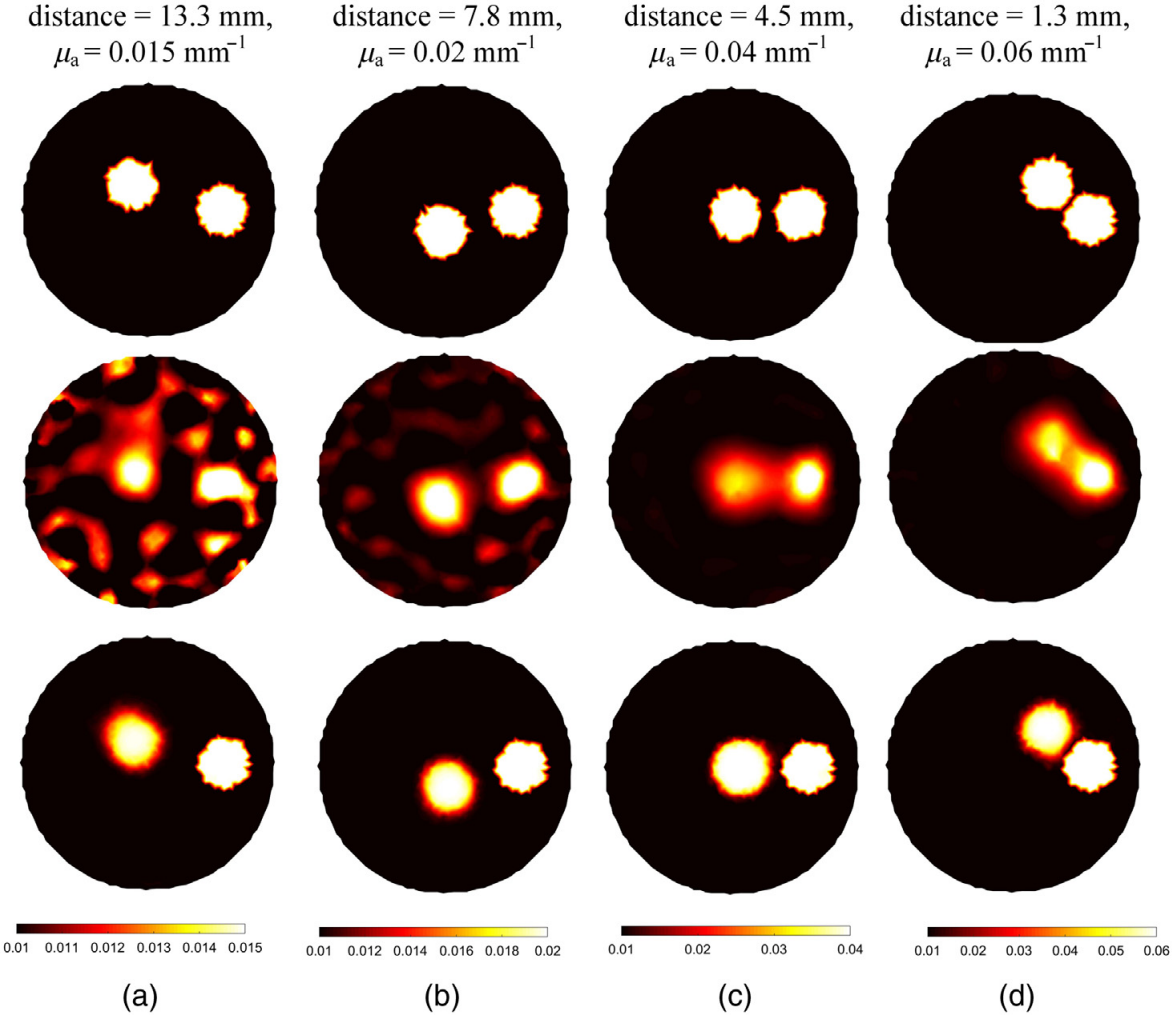
(a)–(d) Examples of reconstructed images for increased intensity of absorption coefficient but with decreased edge-to-edge distance in the case of two inclusions. The first row is the ground truth images. From the second row to the last row, the images are reconstructed using Tikhonov regularization and BPNN, respectively. The edge-to-edge distance and the true values of absorption coefficients for each case are shown at the top of the figure. The reconstructed images of each column are shown at the same scale.

**Fig. 5:**
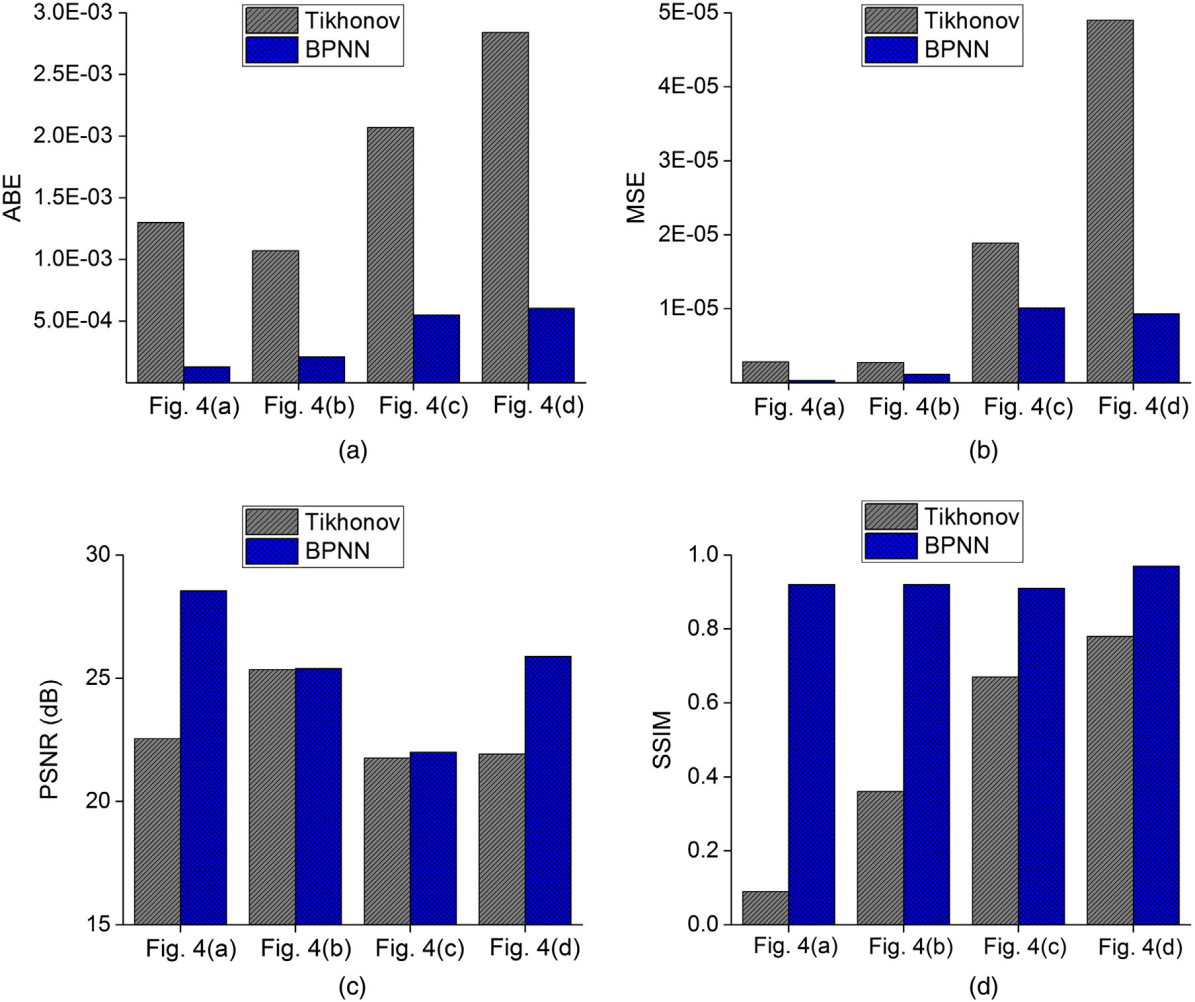
Comparisons of evaluation metrics for reconstructed images of two inclusions using Tikhonov regularization and BPNN. (a) ABE; (b) MSE; (c) PSNR; and (d) SSIM.

Figure 4 shows the capability of BPNN to recover images with two inclusions. And the corresponding quantitative results are compiled in Table 2. The two inclusions are observable and reconstructed with their centers at the correct positions for the two algorithms. But it can be seen that the shapes and the edges of the inclusions can be clearly observed by BPNN even when the edge-to-edge distance is 1.3 mm. However, the absorption coefficients of the inclusions far away from the boundary were underestimated and the images were somehow distorted for the Tikhonov regularization-based reconstruction method. For each evaluation metric, we draw a bar plot of test images in Fig. 5. As the higher accuracy of BPNN can be clearly observed in Fig. 5 and Table 2, we do not compare the cross-sections of different images here. Similar to the previous simulation results of one inclusion, BPNN reduces the artifacts and has offered more than 55.0%, 46.6%, and 16.7% improvement in ABE, MSE, and SSIM, respectively, compared with Tikhonov regularization-based reconstruction algorithm. The PSNRs in Figs. 4(b) and 4(c) are slightly higher than those of the Tikhonov regularization-based reconstruction algorithm because the absorption coefficients reconstructed by Tikhonov regularization have been overestimated with peak values of 0.03 and $0.05\;{\mathrm{mm}}^{-1}$, respectively. By contrast, the peak values obtained by BPNN are 0.02 and $0.04\;{\mathrm{mm}}^{-1}$, which are the same as the true targeted values. For Fig. 4, BPNN can have an average PSNR gain of about 2.56 dB over Tikhonov regularization. Overall, BPNN can obtain better performance.

**Table 2:** The quantitative comparisons in the case of two inclusions presented in Fig. 4.

Metric	Method	Fig. 4(a)	Fig. 4(b)	Fig. 4(c)	Fig. 4(d)
ABE	Tikhonov	$1.30\times 10^{-3}$	$1.07\times 10^{-3}$	$2.07\times 10^{-3}$	$2.84\times 10^{-3}$
	BPNN	$1.28\times 10^{-4}$	$2.10\times 10^{-4}$	$5.50\times 10^{-4}$	$6.04\times 10^{-4}$
MSE	Tikhonov	$2.83\times 10^{-6}$	$2.74\times 10^{-6}$	$1.89\times 10^{-5}$	$4.90\times 10^{-5}$
	BPNN	$3.13\times 10^{-7}$	$1.16\times 10^{-6}$	$1.01\times 10^{-5}$	$9.29\times 10^{-6}$
PSNR (dB)	Tikhonov	22.55	25.35	21.76	21.92
	BPNN	28.55	25.39	22.0	25.88
SSIM	Tikhonov	0.09	0.36	0.67	0.78
	BPNN	0.92	0.92	0.91	0.97

**Fig. 6:**
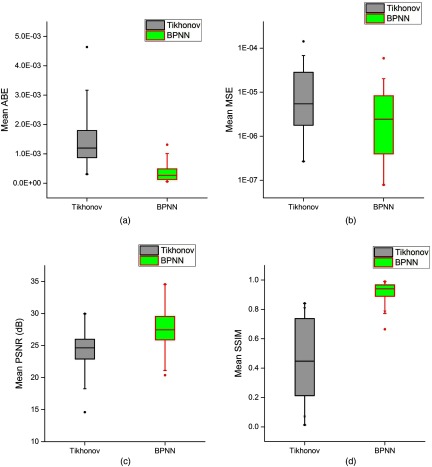
The boxplots for the statistical results (N=1045). (a) ABE; (b) MSE; (c) PSNR; and (d) SSIM.

Here, we reported the results of 1045 samples, which was randomly selected from the dataset to further evaluate the performance of our algorithm. We use the mean and standard deviation (SD) of ABE, MSE, PSNR, and SSIM to evaluate the performances of the two algorithms. The boxplots for the statistical results are presented in Fig. 6. And the corresponding quantitative comparisons are shown in Table 3.

**Table 3:** Mean ± SD of ABE/MSE/PSNR/SSIM for N=1045.

	ABE	MSE	PSNR	SSIM
Tikhonov	$1.50\times 10^{-3}\pm 9.26\times 10^{-4}$	$2.30\times 10^{-5}\pm 3.29\times 10^{-5}$	24.34±2.43	0.46±0.26
BPNN	$3.41\times 10^{-4}\pm 2.50\times 10^{-4}$	$5.97\times 10^{-6}\pm 8.07\times 10^{-6}$	27.79±2.70	0.91±0.06
P-value	<0.001*	<0.001*	<0.001*	<0.001*

*Significant values are marked.

For BPNN, the ABE had a mean value (SD) of $3.41\times 10^{-4}$ ($2.50\times 10^{-4}$); for Tikhonov regularization, the ABE had a mean value (SD) of $1.50\times 10^{-3}$ ($9.26\times 10^{-4}$). The accuracy improvement is 77.3% compared with Tikhonov regularization. The MSE of BPNN has an average of $5.97\times 10^{-6}$, while the average for Tikhonov regularization is $2.30\times 10^{-5}$. The improvement in the average MSE of BPNN with respect to Tikhonov regularization is 74.0%. Furthermore, the average PSNR of BPNN is improved from 24.34 to 27.79 dB compared with Tikhonov regularization. The average value of SSIM (0.91) obtained by BPNN is significant as compared to the average value of SSIM (0.46) produced by Tikhonov regularization. For every one of these metrics, BPNN performs better than Tikhonov regularization being compared.

A student’s t-test is used to determine whether there is statistical significance of the improvement between the evaluation metrics of Tikhonov regularization and BPNN. Significance is achieved at the 95% confidence interval using a two-tailed distribution. The corresponding p-values are also listed in Table 3. Statistically significant differences (p values <0.001) were found in the four evaluation metrics for BPNN versus Tikhonov regularization results, which further confirms that BPNN achieves more stable and effective performance than Tikhonov regularization.

## 4 Discussion and Conclusion

Iterative reconstruction algorithms with regularization have become the dominant approach for solving DOT inverse problem over the past few decades. However, it remains difficult to provide high-quality images. In this study, we explored using a BPNN to recover optical properties to improve the reconstruction accuracy and image quality of DOT. This was motivated by the fact that popular Tikhonov regularization-based reconstruction algorithms tend to produce oversmoothed images, which leads to poor reconstruction accuracy and bad image quality. The superior performance of the proposed algorithm was presented with numerical simulation experiments. Our results indicate that significant improvements of accuracy and image quality can be achieved by the proposed algorithm when compared with the Tikhonov regularization-based algorithm. Qualitative analysis demonstrated that our method can outperform Tikhonov regularization up to 77.3%, 74.0%, 14.2%, and 97.8% in terms of ABE, MSE, PSNR, and SSIM, respectively.

**Fig. 7:**
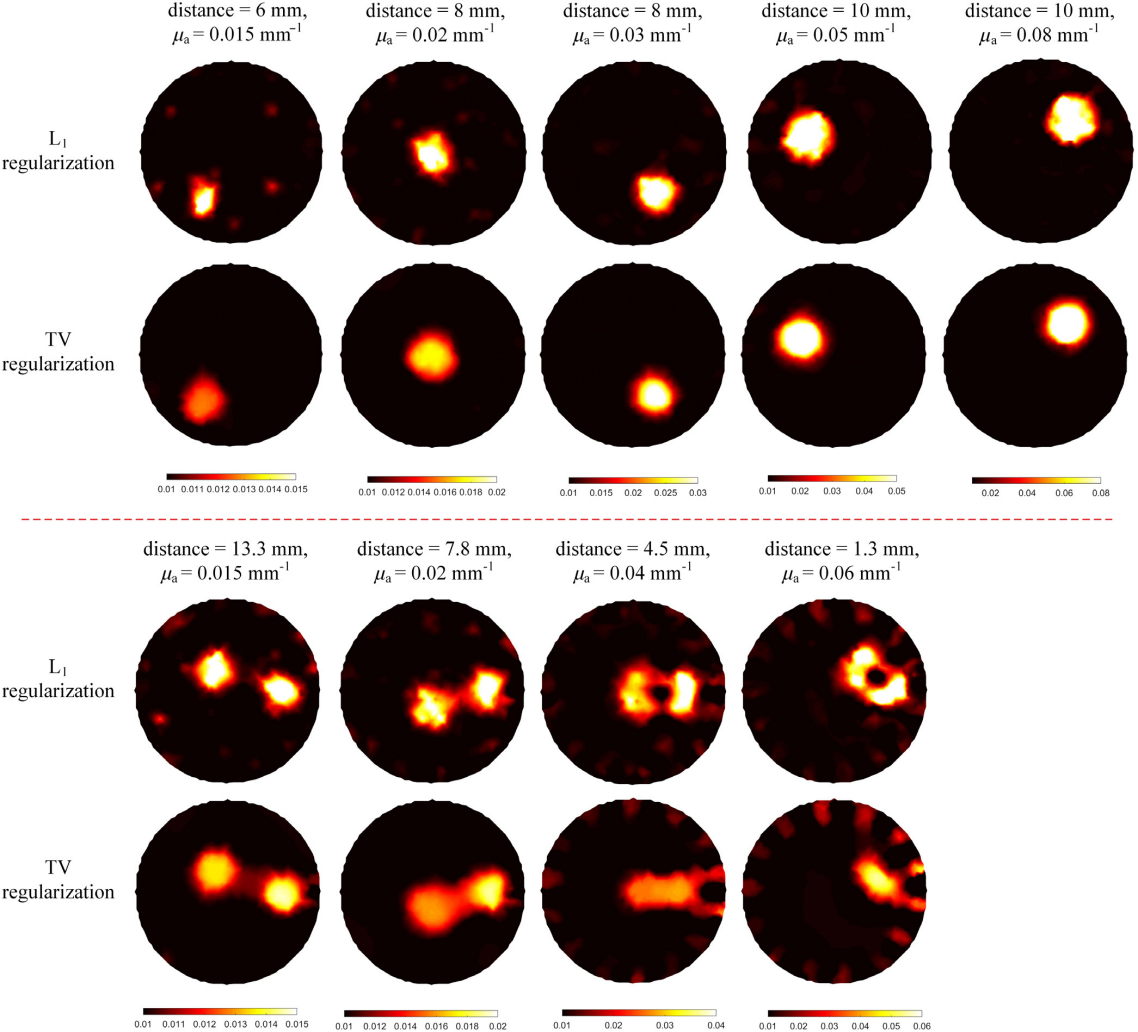
Reconstructed results by both $L_{1}$ and TV regularization algorithms, along with the corresponding reconstructions in Figs. 3 and 4. Top images: reconstructed images with a single inclusion. Bottom images: reconstructed images with two inclusions.

Furthermore, we compared the reconstructed results of the proposed method to those of the $L_{1}$ and TV regularized reconstruction algorithms. The examples of reconstructed images are shown in Fig. 7. For the $L_{1}$ regularized reconstruction algorithm, it was solved with the GPSR algorithm.^46^ For the TV regularized reconstruction algorithm, it was solved by the Split Bregman algorithm.^47^ As for the regularization parameters used in the two reconstruction algorithms, their values were the same and were set to 0.01, which was tuned manually to get the best performance. In the case of single inclusion, the $L_{1}$- or TV-based reconstruction algorithm can obtain better images than the Tikhonov regularization-based reconstruction algorithm in terms of less artifacts. In the case of two inclusions, decreasing the edge-to-edge distance between inclusions leads to degraded image quality for the $L_{1}$- and TV-based reconstruction algorithms. When the edge-to-edge distance of inclusions decreased to 4.5 mm, the two inclusions could not be discriminated for the TV-based reconstruction algorithm. Compared to regularization-based reconstruction algorithms ($L_{2},L_{1}$, and TV), our algorithm performs the best in reconstructing DOT images.

It is possible to obtain high-quality DOT images by training a neural network, even when iterative reconstruction algorithms underperform. Compared to iterative reconstruction algorithms, BPNN-based reconstruction algorithm is capable of: (1) improving the reconstruction accuracy with relatively stable performance; (2) enhancing the image quality with fewer image artifacts in the background; and (3) improving the spatial resolution.

Currently, computational speed is still an open active research area in DOT. To the best of our knowledge, the widely used iterative reconstruction algorithms in DOT require to solve the forward model and to calculate the Jacobian matrix at each iteration.^40^ Therefore, the computational speed is relatively slow. For example, the computational time for each reconstruction in this study is about 1 to 2 min for Tikhonov regularization. For this reason, an algorithm that can fast reconstruct the distribution of optical properties is preferred. Although it takes a rather longer time to train the neuron network for the proposed algorithm, the training is implemented off-line. Once the training is finished, the time for the reconstruction is in a few seconds, which is practically useful for *in vivo* data.

**Fig. 8:**
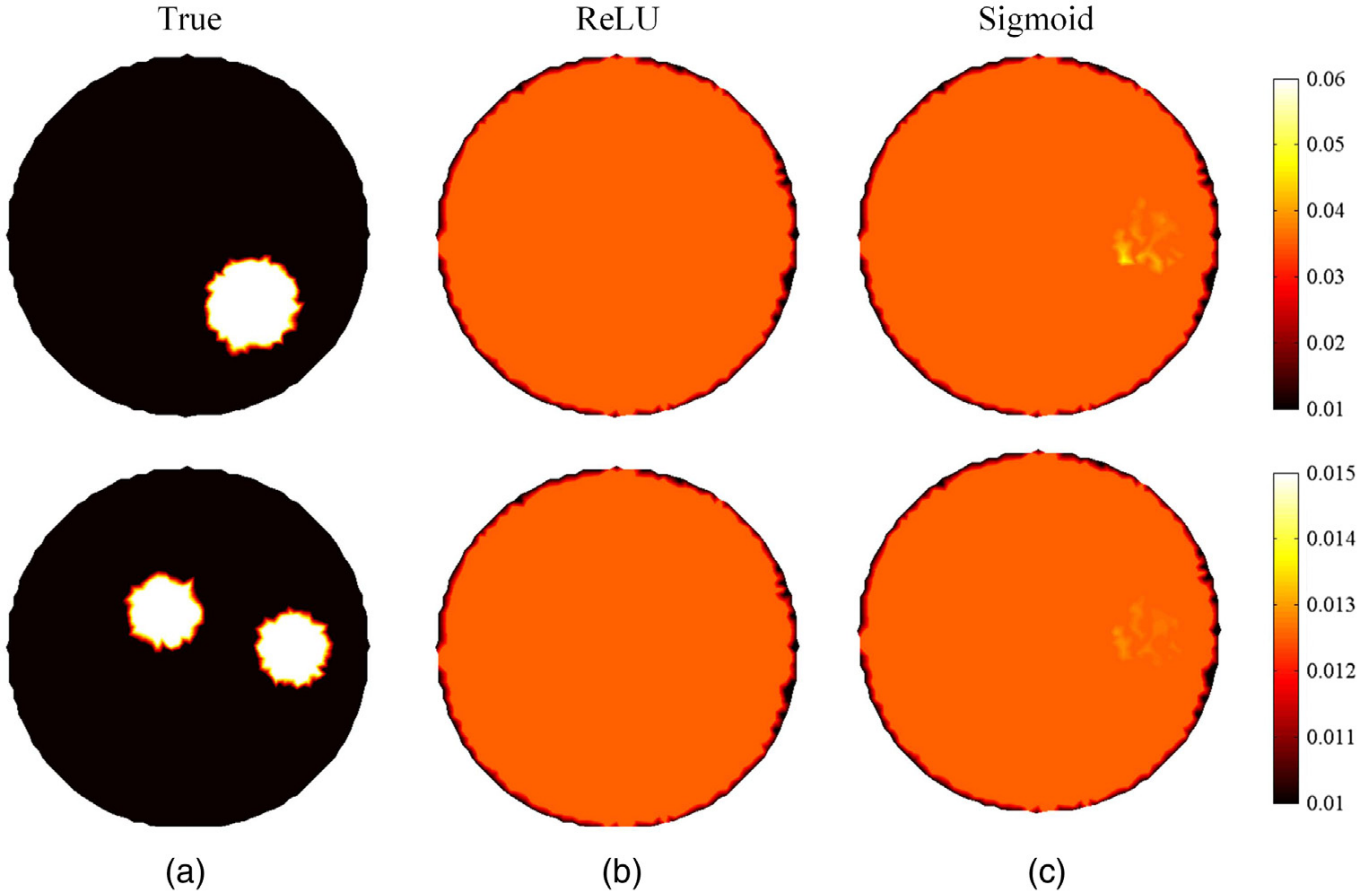
The reconstructed images using different activation functions. (a) True images; (b) and (c) reconstructed images with activation functions of ReLU and sigmoid, respectively.

The effects of different activation functions, including ReLU, Sigmoid, and Tansig, were also examined. The representative results are shown in Fig. 8. Figure 8 shows that the distribution of absorption properties cannot be accurately reconstructed when using either ReLU or sigmoid as the activation function. The mean values of SSIM were 0.02 and 0.03 when using ReLU and sigmoid as the activation function, respectively. There are significant differences between the reconstructed and true images. By contrast, better images can be achieved with the activation function of Tansig and the mean SSIM is 0.91. However, it is still unclear why the activation function of Tansig works for DOT reconstruction.

**Fig. 9:**
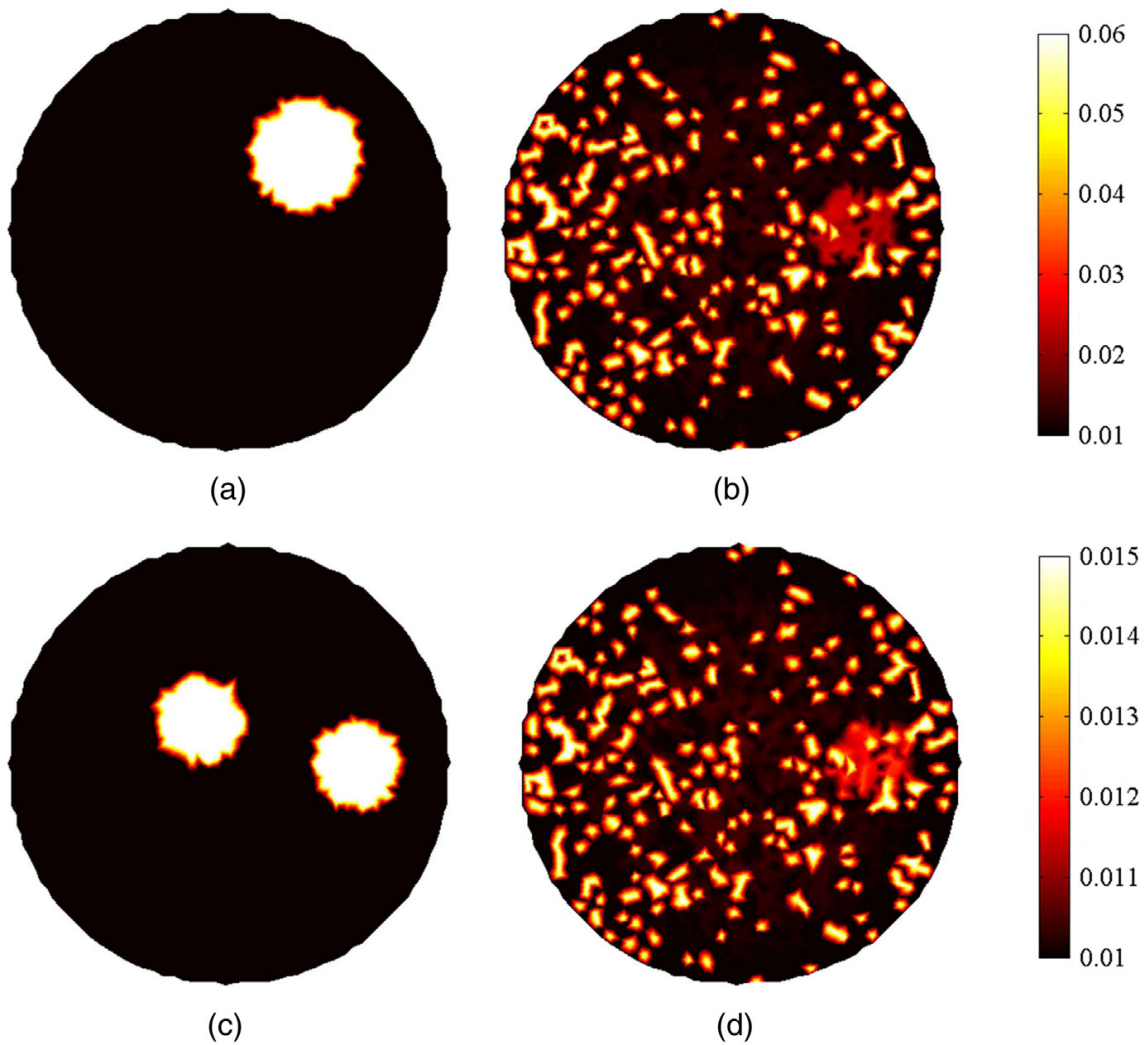
The examples of reconstructed images using the neural network with eight hidden neurons. The first and second rows are the results of single inclusion and two inclusions, respectively. (a) and (c) The true images; (b) and (d) the reconstructed images.

How to determine the number of neurons in the hidden layer needs to be investigated. Except for Eq. (16), one of the typical equations which can be used to determine the number of neurons in the hidden layer is $\log_{2}(n)$,^48^ where n is the number of input neurons. In our experiments, n is 240. Therefore, $\log_{2}(n)$ is about 8. We trained our network with eight hidden neurons. Using the trained network, DOT image reconstruction was performed. The examples of reconstructed images are shown in Fig. 9. Figure 9 shows that the distribution of absorption coefficient cannot be accurately reconstructed. The reason is that using too few neurons in the hidden layer will result in underfitting.

The effects of the learning rate on the performance of BPNN were also analyzed. The learning rate was set to 0.01, 0.1, 1, 10, or 50. The optimal learning rate was the one with the lowest validation loss. In the experiments, the activation function was fixed as Tansig. When the learning rate was set to 50, the validation loss fluctuated over epochs. Therefore, the learning rate of 50 was discarded. The validation losses were 0.15, 0.09, 0.04, and 0.02, corresponding to the learning rates, 0.01, 0.1, 1, and 10, respectively. When learning rate was 10, the lowest validation loss was obtained. We also note that a higher learning rate effectively speeds up the convergence for the training procedure. Therefore, the learning rate was set to 10 in our experiments.

Note that several recent publications have applied various deep learning architectures for solving inverse problems.^49^^–^^51^ For example, a recent work by Sun et al. has shown that a convolutional neural network can be applied to perform image reconstruction under multiple scattering problem in diffraction tomography.^49^ However, our focus is on diffuse optical tomography. Recently, an artificial neural network-based approach is developed to estimate the inclusion location, then the estimated inclusion location is used as a-priori knowledge in DOT reconstruction.^50^ Therefore, the approach is not to reconstruct DOT images directly by learning an artificial neural network. Deep learning technique has been recently applied to reconstruct DOT images, and its superiority has been indicated by comparing with an analytic technique.^51^ The average value of SSIM they reported is 0.46, which is relatively low. By contrast, a higher value of SSIM is obtained by our algorithm, and its value is 0.91. A future study would be to compare the performances of the two algorithms in the same datasets.

Although the proposed BPNN method has achieved promising results, our work could still be considered as a preliminary attempt of applying neural network in DOT; its application in DOT is still very challenging. The performance of BPNN depends on the training data; however, it would be difficult to acquire a sufficient number of real data for training in patient studies. An available strategy is to create training data pairs from breast geometries with known optical properties and the real data is for evaluation. Breast geometries can be obtained from breast MRI images. Since breast geometries have different sizes and shapes, the finite-element meshes generated from breast geometries are different, which leads to different BPNN architectures. To deal with the problem, pixel basis provides a solution.^40^ Each finite-element mesh is mapped to the same pixel basis for network training. Therefore, the trained BPNN will be a universal network. Certain challenges remain which are the subject of further study, including the effect of heterogeneity in the background region as well as performance evaluation using clinical patient data.
